# Ceftaroline Susceptibility among Isolates of MRSA: A Comparison of EUCAST and CLSI Breakpoints

**DOI:** 10.4314/ejhs.v33i1.18

**Published:** 2023-01

**Authors:** Arun Sachu

**Affiliations:** 1 Department of Microbiology Believers Church Medical College, Thiruvalla, Kerala, India

**Keywords:** Ceftaroline, Infection, Susceptibility

## Abstract

**Background:**

Methicillin-resistant Staphylococcus aureus (MRSA) is an important bacterial pathogen causing a number of community-acquired and nosocomial infections. Ceftaroline fosamil is a fifth generation cephalosporin, approved for the treatment of infections caused by MRSA. The main objective of this study was to estimate the susceptibility of ceftaroline among isolates of MRSA by using CLSI and EUCAST breakpoints.

**Materials and Methods:**

Fifty non-duplicate isolates of MRSA were included in the study. Ceftaroline susceptibility was done using E-strip test and interpreted using CLSI and EUCAST breakpoints.

**Results:**

Susceptible isolates were equal (42%) by both CLSI and EUCAST, while resistant isolates were more commonly seen in EUCAST (50%). Ceftaroline MIC ranged from 0.25- >32µg/ml. All the isolates were sensitive to Teicoplanin and Linezolid.

**Conclusions:**

Resistant isolates were less (30%) while using the CLSI 2021 criteria probably due to the inclusion of SDD category. Our study showed that Fourteen isolates (28%) had Ceftaroline MIC >32µg/ml, which is an alarming finding. The high percentage of Ceftaroline resistant isolates in our study probably suggest a hospital spread of Ceftaroline resistant MRSA emphasizing the need for stringent infection control precautions.

## Introduction

Methicillin-resistant Staphylococcus aureus (MRSA) is an important bacterial pathogen causing a number of community-acquired and nosocomial infections, including septicemia, skin and soft tissue infections, osteomyelitis, and endocarditis (1). It is a global health problem and the prevalence of MRSA in India ranges from 25–50% (2). Misuse of Antibiotic has led to high resistance levels in MRSA strains resulting in an increased mortality rate, high costs of care and treatment, and longer periods of hospitalization (3). The emergence of highly virulent community-associated MRSA (CA-MRSA) causing skin infections, sepsis, toxic shock syndrome, and necrotizing pneumonia is a major concern (4). The drug of choice for treating severe MRSA infections is Vancomycin. However, its use has unfortunately been associated with several limitations like poor drug penetration into the tissues, narrow therapeutic index, slow bactericidal activity, difficulty in achieving pharmacokinetic and pharmacodynamic targets and potential side effects like nephrotoxicity and ototoxicity (5,6). There are also reports of treatment failures with vancomycin in critically ill patients due to suboptimal therapeutic levels or high MIC(Minimum Inhibitory Concentration) values (7). Alternative drugs like Teicoplanin, linezolid and daptomycin are being increasingly used for treatment of MRSA infections (8).

MRSA is frequently isolated from complicated skin and soft tissue infections and the cases of MRSA are increasing among outpatients (9,10,11,12). Ceftaroline fosamil is a fifth generation cephalosporin active against methicillin-susceptible (MSSA) and MRSA. It was approved in October 2010 by the United States Food and Drug Administration (US FDA) for the treatment of adults with community-acquired bacterial pneumonia and acute bacterial /skin and skin structure infections (ABSSSI)(13). Ceftaroline acts by inhibiting cell wall synthesis by binding to Penicillin Binding Proteins (PBP) 1, 2, 3 and PBP 2a for MRSA(14). Studies have shown that the drug is well tolerated by patients and is as effective as vancomycin, daptomycin and linezolid in eradicating MRSA (15,16). Resistance to ceftaroline is uncommon but several studies have reported decreased susceptibility of MRSA to ceftaroline in sporadic cases. The resistance may be due to the mutation within PBP 2a protein, in particular, outside the Penicillin-Binding Domain (nPBD) (17,18).

Ceftaroline has been approved for the treatment of cSSTI (Complicated Skin and Soft tissue infections) at a standard dosage of 600 mg every 12 h (given over 60 min) in adults (19). In 2017, the European Medicines Agency approved a higher dosing regimen of ceftaroline (600 mg every 8 h over 120 min) for cSSTI caused by S. aureus with a ceftaroline MIC of 2 or 4 mg/L. European Committee on Antimicrobial Susceptibility Testing (EUCAST) introduced an intermediate breakpoint of 2 mg/L for ceftaroline against S. aureus, for indications other than pneumonia, in version 8.0 of the EUCAST breakpoints due to the approval of a higher dosing regimen by the European Medicines Agency. As a result, the EUCAST resistant breakpoint for ceftaroline against S. aureus increased from >1 mg/L in version 7.1 of the breakpoint tables to >2 mg/L in version 8.0(20,21,22).

Clinical Microbiology laboratories in several countries use breakpoints published by CLSI (Clinical Laboratory Standards Institute) for susceptibility testing. In January 2019, CLSI also modified the ceftaroline breakpoints and introduced the susceptible dose dependent (SDD) category for this agent, based on the recommendation by the European Medicines Agency in 2017 (High dose regimen of 600 mg every 8 h over 120 min), although this dosing regimen is not approved by the US Food and Drug Administration(23). There are very few studies in India evaluating the susceptibility of *S.aureus* to ceftaroline and there is very little data available about the susceptibility pattern of *S.aureus* to ceftaroline (24,25,26). The aim of this study was to:

1. Estimate the rate of in vitro susceptibility of ceftaroline among isolates of MRSA by E-test strip using CLSI and EUCAST breakpoints;
2. To assess the agreement between the two guidelines for susceptibility testing of Ceftaroline; and
3. To find the antimicrobial susceptibility pattern of MRSA isolated during the study period.

## Materials and Methods

This prospective study was conducted in the Department of Microbiology over a period of 9 months from April 2021 to December 2021 after obtaining clearance from the Ethical Committee. Fifty non-duplicate isolates of Methicillin Resistant *S. aureus* strains isolated from various clinical samples were included in the study. Isolates which showed gram positive cocci in clusters on grams stain and gave positive results on catalase, slide and tube coagulase where identified as *S. aureus*.

Screening for methicillin resistance was done by modified Kirby Bauer disc diffusion method using cefoxitin (30 µg) discs. A zone size of ≥22 mm was interpreted as methicillin sensitive and ≤21 mm was interpreted as methicillin resistant as per Clinical and Laboratory Standards Institute (CLSI) guidelines (23). S. aureus American Type Culture Collection (ATCC) strain 25923 and S. aureus ATCC strain 43300 were used as susceptibility and resistance controls respectively. Isolates which were Methicillin sensitive were excluded from the study. Antimicrobial susceptibility of the isolates was done by Kirby Bauers disc diffusion

Testing for ceftaroline susceptibility was done by E- test strip method. The ceftaroline E-test strips (0.002–32 µg/mL) was obtained from Biomerieux, France. The E- test strips were placed on the lawn culture of the organism and the plates were incubated at 37°C for 18–24 hours. MIC's were read where the ellipse intersects the MIC (Minimum Inhibitory Concentration) scale. Since E-test strip has continuous gradient, MIC values “in-between” two-fold dilutions can be obtained. These values were rounded up to next two-fold dilution before categorisation. MICs were interpreted according to EUCAST version 11.0 and CLSI 2021.

**EUCAST** version 11.0 for Ceftaroline (In pneumonia) - ≤1- Susceptible, > 1- Resistant

**EUCAST** version 11.0 for Ceftaroline (For conditions other than pneumonia)-≤1- Susceptible, >2- Resistant

**CLSI 2021**- ≤1- Susceptible, 2–4(Susceptible Dose Dependent), ≥8- Resistant.

## Results

Among the 50 isolates of MRSA, 26 isolates were from samples received from the Surgery department, while 18 and 6 were from Medicine and Obstetrics departments respectively. Of the 50 patients, 30 were admitted in the ward while ten patients were admitted in Intensive care. The remaining ten samples were from patients attending the Outpatient Department. Among the 50 isolates of MRSA, Ceftaroline susceptibility by CLSI and EUCAST (Table 1).

**Table 1:** Ceftaroline Susceptiblity by CLSI and EUCAST

Reference	S,n(%)	I,n(%)	SDD,n(%)	R,n(%)
CLSI 2021	21(42)	NA	14(28)	15(30)
EUCAST 11.0	21(42)	4(8)	NA	25(50)

S-Susceptible, I- Intermediate, R- Resistant, SDD- Susceptible Dose Dependent, NA- Not Applicable, CLSI- Clinical Laboratory Standards Institute, EUCAST- European Committee on Antimicrobial Susceptibility Testing.

**Figure 1:**
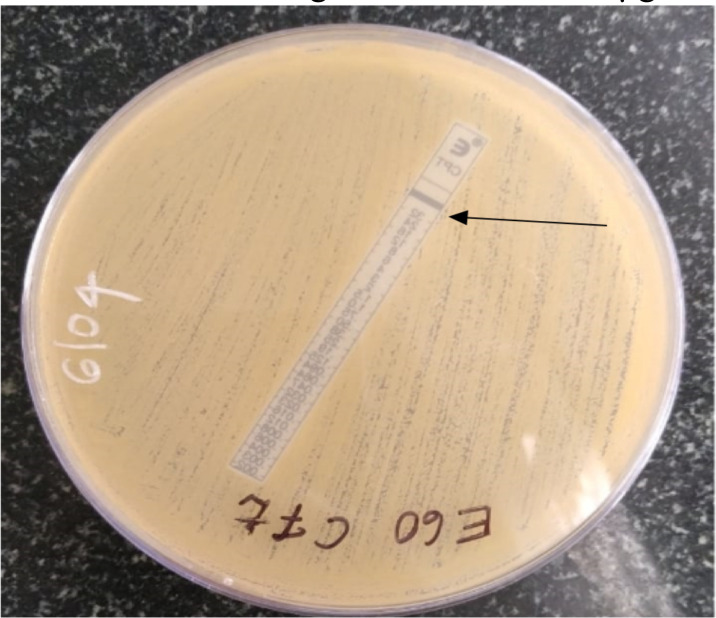
E-test method for Ceftaroline susceptibility (arrow= E test MIC > 32µg/ml).

Susceptible isolates were equal (42%) by both CLSI and EUCAST, while resistant isolates were more commonly seen in EUCAST (50%). Ceftaroline MIC ranged from 0.25- >32µg/ml. An Isolate of MRSA with Ceftaroline MIC >32µg/ml (Fig. 1). Twenty-one isolates were susceptible by both CLSI and EUCAST and had MIC ranging from 0.25–0.50 µg/ml (Table 2).

**Table 2:** MIC distribution of the isolates

MIC (µg/Ml)	No of isolates (%)
0.25	6(.12)
0.38	7(.14)
0.5	8(.16)
2	5(.1)
4	9(.18)
12	1(.02)
>32	14(.28)

MIC- Minimum inhibitory Concentration, Ceftaroline MIC of ATCC strain 25923 and ATCC strain 43300 was 0.25 µg/ml and 0.38µg/ml respectively.

Fourteen isolates (28%) had Ceftaroline MIC >32µg/ml. The MIC50 of the isolates was 2µg/ml. Antimicrobial susceptibility pattern of the isolates (Table 3). All the isolates were sensitive to Teicoplanin and Linezolid. Majority of the isolates were also sensitive to Gentamicin, Tetracycline and Cotrimoxazole.

**Table 3:** Antimicrobial susceptibility pattern of MRSA isolates

Antimicrobial agent	Sensitive n(%)	Intermediate n(%)	Resistant n(%)
Gentamicin	43(86)	2(4)	5(10)
Ciprofloxacin	12(24)	10(20)	28(56)
Erythromycin	10(20)	7(14)	33(66)
Clindamycin	25(50)	1(2)	24(48)
Tetracycline	43(86)	1(2)	6(12)
Teicoplanin	50(100)	nil	nil
Linezolid	50(100)	nil	nil
Cotrimoxazole	41(82)	3(6)	6(12)

## Discussion

*Staphylococcus aureus* is an important cause of nosocomial as well as community acquired infections. Treatment of Infections caused by MRSA is very challenging and the emergence of Multidrug resistant MRSA has made the management of MRSA Infections even more difficult (24). Ceftaroline has been approved as alternative for infections caused by MRSA. The main focus of this study was to see the effects on Ceftaroline susceptibility by using different guidelines.

In this study 30% of the isolates were Ceftaroline resistant by using the CLSI 2021 guidelines, while 50% were resistant using the EUCAST 11.0 guidelines. The reduction in number of resistant isolates while using CLSI 2021 criteria was due to the SDD (2-4µg/ml) criteria in the 2021 guidelines. Ceftaroline susceptibility among MRSA in Different studies is shown in Table 4. This study has reported high Ceftaroline resistance among MRSA when compared to other studies (Table 4). Ceftaroline resistance of 30% was found while using CLSI 2021 criteria which is similar to the study conducted by Andrey et al who reported Ceftaroline resistance of 24% (29). Study conducted by Dehkordi *et al.* on antibiotic resistance pattern of the MRSA isolated from hospital food showed that 37 MRSA isolates obtained from 485 samples were resistant to Ceftaroline (30). Another study conducted by Abdolmaleki *et al* showed that 36 out of 65 S. aureus from external washings of samples of hospital cockroaches and 15 out of 37 S. aureus isolated from gut content of hospital cockroaches were MRSA (31). A study conducted by Zhang et al showed that the rate of resistance to ceftaroline among MRSA isolates was much higher in the Asia-Pacific region compared to other parts of the world (32). Study conducted by Bakthavatchalam YD *et al* showed that that 6% and 2% of tested S. aureus isolates had a ceftaroline MIC of 2 µg/mL and 4 µg/mL, respectively which according to the old CLSI guidelines were interpreted as intermediate and resistant respectively. If the current CLSI guidelines are applied, these isolates will be classified as SDD and not as resistant. Bakthavatchalam YD *et al* reported a total of 13 isolates with ceftaroline MIC 2–4 µg/mL, while our study reported 14 isolates in the SDD category (2–4 µg/mL) while using CLSI 2021 criteria (25).

**Table no 4:** Ceftaroline susceptibility among MRSA in Different studies

Authors	Number of isolates	Ceftaroline Susceptiblity(%)	Criteria Used
Bakthavatchalam YD et al (25)	86	84.88	CLSI 2013
Gaikwad et al (26)	28	93.33	CLSI 2015
Flamm RK et al (27)	1072	98.4	CLSI 2011
Khoshbayan et al (28)	228	97.3	CLSI 2019

Our study showed that Fourteen isolates(28%) had Ceftaroline MIC >32µg/ml, while one isolate had Ceftaroline MIC-12µg/ml. Long et al reported PBP2a mutations causing high level Ceftaroline resistance (MIC>32µg/ml) among isolates of MRSA(33). Studies have shown that the majority of MRSA isolates with ceftaroline MIC values of 2µg/ml had a single amino acid substitution in the non-penicillin-binding domain of penicillin-binding protein 2a (PBP2a), and isolates with ceftaroline MICs of 4 µg/mlor 8 µg/ml, all had an additional single amino acid substitution in the penicillin-binding domain of PBP2a(34,35,36). MIC50 of the isolates in the studies conducted by Gaikwad and Basireddy et al were 0.38µg/ml and 0.5µg/ml respectively which was discordant with the findings in our study, which showed an MIC 50 of 2 µg/ml (24,26).

The MIC distribution of the isolates in our study were discordant with the study conducted in Turkey which found that 94.3% of tested MRSA isolates were inhibited by ceftaroline (MIC≤1 µg/mL) (37). In our study all the 50 isolates(100%) were susceptible to Teicoplanin and Linezolid, while the resistant rates to Erythromycin, Clindamycin and Ciprofloxacin were 66%, 48% and 56% respectively. This finding was quite similar to the study conducted by Elfeky et al who showed that 63% of the isolates were resistant to Erythromycin, Clindamycin and Ciprofloxacin(38). Susceptiblity pattern of the 14 isolates with Ceftaroline MIC >32µg/ml were not completely identical. Among these 14 isolates, 85.7% were sensitive to Gentamicin and Tetracycline, while 21.4% were sensitive to Erythromycin and Ciprofloxacin. In addition, 71.4% of these isolates were sensitive to Cotrimoxazole, while 35.7% of these isolates were susceptible to Clindamycin. Among the 14 isolates, 50% were from patients admitted in surgical department, while 42.9% were from patients in general medicine department.

In conclusion, in this study Ceftaroline susceptibility in MRSA was evaluated using CLSI and EUCAST. Only 42% of the isolates were Ceftaroline susceptible by both guidelines. Resistant isolates were less (30%) while using the CLSI 2021 criteria probably due to the inclusion of SDD category. Ceftaroline MIC ranged from 0.25- >32µg/ml. Our study showed that Fourteen isolates(28%) had Ceftaroline MIC >32µg/ml, which is an alarming finding. The high percentage of Ceftaroline resistant isolates in our study probably suggest a hospital spread of Ceftaroline resistant MRSA emphasizing the need for stringent infection control precautions. To the best of our knowledge this is the first study from India showing a high percentage of Ceftaroline resistance, probably suggesting that Ceftaroline is not a good treatment option for MRSA infections in our hospital.

The study had a relatively small sample size .Genome sequencing of the isolates could not be done to look for any mutations in PBP2a. Epidemiologic characterization of the isolates could not be done to look for any clonal transmission.
